# Severe Anxiety Post-COVID-19 Infection

**DOI:** 10.1155/2021/9922508

**Published:** 2021-12-01

**Authors:** Maggie Driscoll, Jason Gu

**Affiliations:** Department of Psychiatry, Lehigh Valley Health Network, Bethlehem, Pennsylvania, USA

## Abstract

COVID-19 infection is linked to increased risk of neuropsychiatric symptoms such as psychosis and suicidal ideation/behavior. After further review of the literature, there is not a large body of data on anxiety following COVID-19 infection. Most literature found is related to fear/anxiety of contracting and dying from COVID-19. We illustrate a case of a 27-year-old male with no previous psychiatric treatment history or symptomology, who developed severe anxiety with intrusive thoughts of self-harm via firearm after COVID-19 infection. Given the severe nature of the anxiety and intrusive thoughts, the patient feared for his safety and sought acute inpatient admission. The patient was effectively treated with group therapy and psychotropic medications and was able to be discharged in a timely manner with outpatient psychiatric follow-up. Much is still unknown of COVID-19. With this case report, we discuss a potential relationship between anxiety and COVID-19 infection.

## 1. Introduction

Much is still unknown of COVID-19, much less of any potential psychiatric sequelae of COVID-19. The majority of studies focus more on anxiety related to fear of the pandemic [1], but few look at anxiety as a potential sequela of COVID-19. Studies have shown that the COVID-19 virus is effective in entering the central nervous system [2–4]. Therefore, some have postulated that anxiety could be a long-term sequela of COVID-19 infection [5]. Few studies exist on this relationship. A case report was found of patients experiencing severe anxiety in the context of an active, new COVID-19 infection [6]. Few case reports exist regarding neuropsychiatric symptoms after COVID-19 infection. A case report was found of a 52-year-old patient with no past psychiatric history who developed psychosis after infection of COVID-19 [7]. Even fewer studies exist on sequelae of mild COVID-19 cases. A recent retrospective cohort links severe COVID-19 infection with increased incidence of psychiatric symptom [8]. We present a case of a 27-year-old male with no previous psychiatric treatment history or symptomology, who developed severe anxiety with intrusive thoughts of self-harm via firearm after a mild case of COVID-19.

## 2. Case Presentation

Mr. M is a 27-year-old male with no significant past medical or psychiatric history. He presented to the emergency department due to worsening mood, anxiety, and intrusive thoughts of shooting himself. An emergency psychiatric clinician completed a Columbia Suicide Rating Scale in which the patient responded “yes” to 3 of the 6 questions. Mr. M reported “yes” to the following questions: (1) Have you actually had any thoughts of killing yourself? (2) Have you been thinking about how you might do this? (3) Have you had these thoughts and had some intention of acting on them? After the evaluation was completed, acute inpatient psychiatric treatment was recommended. Mr. M was subsequently admitted to the inpatient psychiatric unit voluntarily. On admission, he reported a history of COVID-19 infection roughly 4 weeks prior, with subsequent acute onset anxiety 1 day prior to hospitalization. He stated he was having intrusive thoughts of putting his loaded handgun to his head. He denied intent but stated he felt very unsafe with the firearm in his home due to these intrusive images. He stated he wanted to go upstairs where the firearm was stored to remove the ammunition from the gun but felt unsafe to do so. He reported that the thoughts were strong enough in intensity that he feared for his safety in his own home. He contacted his mother who removed the firearm from the home and urged the patient to seek psychiatric treatment. At the time of admission, Mr. M denied symptoms of psychosis, mania, depression, or PTSD. During initial presentation and throughout hospital stay, Mr. M adamantly denied suicidal ideation, intent, or plan to harm himself. Regarding substance use, he reported vaping nicotine. He also reported alcohol use on the weekends with friends drinking up to 6 drinks per night with up to 18 drinks total for a weekend. Mr. M denied any illicit drug use. Of note, he denied any previous psychiatric treatment history. He was naïve to psychotropic medications. As per collateral history obtained from the patient's wife, she confirmed that the patient had no previous psychiatric treatment history and his anxiety symptoms were preceded by a COVID-19 infection 4 weeks prior to admission. Mr. M had a family history significant for depression and anxiety in his mother. No suicide attempts or substance abuse family history was noted. He is married with no children. He attained a college degree and works for a local insurance company. Vital signs on admission and during hospital stay were unremarkable. Laboratory workup in the emergency department was also unremarkable. No imaging was performed. Mr. M was treated with hydroxyzine 25 mg q 6 hours prn for anxiety and melatonin 3 mg nightly prn for sleep, as well as fluoxetine, which was titrated up from 10 mg to 20 mg upon discharge, for anxiety and intrusive thoughts. Through the course of the hospitalization, Mr. M was stabilized with a multidisciplinary approach that included daily lethality assessments, pharmacotherapy, and group psychotherapy over a 4-day hospital stay.

## 3. Discussion

Our case illustrates an otherwise healthy man who developed sudden and severe anxiety. The only notable stressor is a recent COVID-19 infection. Given available but limited evidence associating COVID-19 infection with neuropsychiatric symptoms, we ask if our patient's anxiety could be related to his COVID-19 infection.

One way to help understand the sequelae of the current COVID-19 infection is to compare psychiatric symptoms seen in COVID-19 to past coronavirus infections like severe acute respiratory syndrome (SARS), which was shown to be associated with new psychiatric diagnoses on follow-up including posttraumatic stress disorder, depression, somatoform pain disorder, panic disorder, and obsessive-compulsive disorder [9]. Though anxiety is not specifically listed, our patient's presentation could be classified as a panic attack or obsessions. One limitation of this case was that the anxiety occurred prior to admission and had resolved by the time the patient was admitted to inpatient psychiatry, and thus, we were not able to evaluate the anxiety as it occurred and were limited to patient's history and collateral history. This made it difficult to further narrow down the patient's diagnosis.

This case parallels other case reports in some ways in that our patient experienced extreme anxiety and suicidal thoughts like the patients seen by Ferrando et al. and Chacko et al. in their respective case reports [6, 7]. However, our patient exhibited no psychosis and his anxiety quickly abated when the firearm was removed from his home. His anxiety occurred several weeks after his COVID-19 infection, and he did not experience any further symptoms while being treated on inpatient psychiatry. The acute onset and resolution of our patient's symptoms, while reassuring from a treatment perspective, are also concerning for how quickly and severely these affected our patient and offer significant concern for potential other patients who do not have close available supports to help secure items like firearms that can be lethal in suicide. Thus, further research is needed to assess neuropsychiatric sequelae of COVID-19 infections.

As a case report, possible conclusions are limited. However, should further study confirm anxiety as a sequela of COVID-19 infections, the implications could be significant. Not only would that help further our understanding of COVID-19 but also that practically would help us to focus efforts to screen and treat a potentially vulnerable population.

## 4. Conclusion

As the COVID-19 pandemic progresses, more is being learned about its effects and aftereffects. Our case mirrors others that describe potential neuropsychiatric symptoms related to COVID-19 infection. In our case, our patient after COVID-19 infection experienced severe anxiety that was debilitating and dangerous enough to require psychiatric admission. Given the potential psychiatric severity of this presentation in the absence of other precipitating factors, we recommend further study into anxiety as a potential sequela of COVID-19 infection.

## Data Availability

The authors confirm that the data supporting the findings of this case report are available within the article.

## References

[1] Choi E. P., Hui B. P., Wan E. Y. (2020). Depression and anxiety in Hong Kong during COVID-19. *International Journal of Environmental Research and Public Health*.

[2] Netland J., Meyerholz D. K., Moore S., Cassell M., Perlman S. (2008). Severe acute respiratory syndrome coronavirus infection causes neuronal death in the absence of encephalitis in mice transgenic for human ACE2. *Journal of Virology*.

[3] Baig A. M., Khaleeq A., Ali U., Syeda H. (2020). Evidence of the COVID-19 virus targeting the CNS: tissue distribution, host–virus interaction, and proposed neurotropic mechanisms. *ACS Chemical Neuroscience*.

[4] Ding Y., He L., Zhang Q. (2004). Organ distribution of severe acute respiratory syndrome (SARS) associated coronavirus (SARS-CoV) in SARS patients: implications for pathogenesis and virus transmission pathways. *The Journal of Pathology*.

[5] Rogers J. P., Chesney E., Oliver D. (2020). Psychiatric and neuropsychiatric presentations associated with severe coronavirus infections: a systematic review and meta-analysis with comparison to the COVID-19 pandemic. *The Lancet Psychiatry*.

[6] Ferrando S. J., Klepacz L., Lynch S. (2020). COVID-19 psychosis: a potential new neuropsychiatric condition triggered by novel coronavirus infection and the inflammatory response?. *Psychosomatics*.

[7] Chacko M., Job A., Caston F., George P., Yacoub A., Cáceda R. (2020). COVID-19-induced psychosis and suicidal behavior: case report. *SN Comprehensive Clinical Medicine*.

[8] Taquet M., Luciano S., Geddes J. R., Harrison P. J. (2021). Bidirectional associations between COVID-19 and psychiatric disorder: retrospective cohort studies of 62 354 COVID-19 cases in the USA. *The Lancet Psychiatry*.

[9] Lam M. H.-B., Wing Y.-K., Yu M. W.-M. (2009). Mental morbidities and chronic fatigue in severe acute respiratory syndrome survivors. *Archives of Internal Medicine*.

